# Prevalence of tobacco use and passive exposure among adolescent athletes aged 13–14 years in Türkiye: A cross-sectional study

**DOI:** 10.18332/tid/202181

**Published:** 2025-03-24

**Authors:** Tuğba Kocahan, Erdoğan Asar, Aydan Örsçelik, Çağrı E. Şahin, Gökhan Büyüklüoğlu, Buse Ataoğlu, Yunus E. Bulut, Toker Ergüder

**Affiliations:** 1 Department of Sports Medicine, Gulhane Faculty of Medicine, University of Health Sciences, Ankara, Türkiye; 2 Department of Medical Informatics, Gulhane Faculty of Medicine, University of Health Sciences, Ankara, Türkiye; 3 Department of Public Health, Gulhane Faculty of Medicine, University of Health Sciences, Ankara, Türkiye

**Keywords:** adolescent, athlete, tobacco use, passive smoking

## Abstract

**INTRODUCTION:**

Youth smoking and passive exposure is a serious public health problem. This study examined the prevalence of tobacco use and passive exposure to smoke among adolescent athletes.

**METHODS:**

This cross-sectional study was conducted in Ankara, Türkiye, in 2023. It was based on the survey data from 201 licensed athletes, of whom 36.3% were female and 63.7% were male, aged 13–14 years, residing in Ankara province. The respondents were asked to complete a questionnaire regarding their use of tobacco products and exposure to secondhand smoke.

**RESULTS:**

The mean age of the respondents was 13.4 ± 0.5 years, with a mean sports age of 3.2 ± 2.0 years. Among the athletes, 92% (n=185) reported they never used tobacco products, 7.0% (n=14) tried, 0.5% (n=1) quit using tobacco products, and 0.5% (n=1) currently smoke tobacco. Among the adolescents, 59.2% reported passive exposure to tobacco smoke inside and 71.1% outside the home. The rates of daily secondhand smoking (≥1 h or <1 h) did not differ by gender (p>0.05), but weekly passive exposure was significantly higher in male athletes (67.0% vs 33.0%, p<0.05), as was no exposure (female: 34.1%, male: 65.9%, p<0.05). Among all athletes, 46.7% reported that at least one of their parents smoked, while 19.9% reported that both parents smoked.

**CONCLUSIONS:**

Tobacco use among adolescent athletes in Türkiye is minimal, yet passive smoking is a significant concern. Given the high prevalence of passive smoke exposure reported among adolescent athletes, particularly at home and in outdoor settings, further attention to reducing such exposure is warranted. The low prevalence of active tobacco use in this population suggests that preventive measures may be effective in maintaining low smoking rates as these adolescents age. Significantly lower prevalence of active smoking among the respondents could be attributed to their active engagement in sports and their young 13–14 years age.

## INTRODUCTION

The use of tobacco products and exposure to secondhand smoke represents a significant public health concern, with adverse effects on both individual and public health. It is estimated that over 8 million deaths are attributed to direct tobacco use, and approximately 1.3 million to passive tobacco smoking among individuals who do not smoke^1^. The scientific evidence unequivocally demonstrates that there is no safe level of exposure to secondhand tobacco smoke. The World Health Organization (WHO) and other leading health organizations concur that passive exposure to tobacco smoke causes a range of serious and often fatal diseases, including lung and other cancers, as well as cardiovascular and respiratory diseases. Furthermore, exposure to secondhand smoke can have adverse effects on children, including fetuses and newborns^1^.

The extant literature demonstrates that adolescents who are passively exposed to tobacco smoke are more likely to experience symptoms related to respiratory tract infections, including shortness of breath, difficulty in exercising, wheezing during or after exercise, and a dry cough at night. Additionally, they are more likely to be unable to attend school due to illness, to experience difficulty in exercising, and to exhibit depressive symptoms^2,3^. Adolescents residing with active smokers and exposed to passive cigarette smoke for a minimum of one hour per day are more prone to seek emergency department care^2^. Children exposed to parental smoking are at an elevated risk for subclinical atherosclerosis^4^, death from coronary heart disease in adulthood^5^, and deteriorated bone health^6^.

Both active tobacco use and passive smoking result in an elevation of metabolic syndrome and cardiometabolic risk factors in adolescents^4,5^. This phenomenon persists beyond adolescence, with adverse health consequences continuing into adulthood. Furthermore, passive exposure to tobacco smoke during adolescence increases the likelihood of subsequent active tobacco use^7^. It has been recommended that the issue of combating tobacco use in pediatric age groups and adolescents should be addressed with caution; as young athletes are commonly used as examples to follow or sources of inspiration and motivation for other population groups. They can also be used as trendsetters, hence, their health behaviors matter^8-10^.

Despite its acknowledged detrimental impact on public health, active and passive tobacco smoking among adolescents persists globally. Yet, it is a preventable problem^1^. In order to address the global issue of tobacco use, the WHO has developed a series of policies and strategies under the WHO Framework Convention on Tobacco Control (WHO FCTC). Türkiye ratified the WHO FCTC in 2004. Currently, Türkiye is striving to safeguard its population at the highest level through the implementation of comprehensive measures, including smoking bans in indoor areas and public transport vehicles, the display of deterrent health warnings on cigarette packages, the increases in taxes on tobacco products, and the dissemination of public awareness campaigns^11^. These practices represent a significant advancement in the global effort to combat tobacco use. However, further action is required to prevent both active and passive tobacco consumption, particularly among specific demographic groups, including adolescents and athletes.

Limited research exists on smoking habits among licensed adolescent athletes in Türkiye^12,13^. Prior studies have documented varying rates of tobacco use and passive smoke exposure among adolescents globally. For instance, a global study of youth aged 12–15 years reported a secondhand smoke exposure prevalence of 55.9%^14^. In Türkiye, studies have shown that 21.72% of male students aged 14–18 years were cigarette smokers^12^, while another study among students aged 11–19 years revealed a smoking rate of 28.1%^13^. However, the prevalence of tobacco use among adolescent athletes, particularly those aged 13–14 years, remains understudied. The objective of this study was to assess tobacco use and passive exposure to tobacco smoke among a sample of adolescent athletes in Türkiye and to examine their characteristics and associated factors.

## METHODS

### Study design

This research is a descriptive cross-sectional study of licensed young athletes aged 13–14 years from the central districts of Ankara, the capital city of Türkiye. The study was conducted between 1 April and 31 May 2024, following the approval of the Ethics Committee (dated 27 February 2024, and registration number 2024/93) from the Health Sciences University, Gulhane Scientific Research Ethics Committee. Athletic centers were visited between 1 April and 31 May 2024.

The power analysis of the study was conducted using OpenEpi (version 3.1.9.7) software. The number of athletes in the sample group was determined to be 167 with a 95% confidence level (5% error level), a population size of 7476 (the number of active athletes in Ankara in January 2024), an expected rate (p) of smoking among young people aged 13–15 years was taken as 7.7%^15^, and a deviation level (d) of 4%. A sample size of 201 was obtained by adding 20% to the values above, which accounts for potential cases of withdrawal from the research, missing data, and other factors based on expert opinion. The central districts of Ankara were selected as the sample group, and the number of athletes to be included in the study was determined using districts as clusters for sampling.

Written and verbal permission to conduct the survey was obtained from the relevant sports clubs. In order to be included in the study, athletes had to meet the following criteria: to be aged 13–14 years, a licensed athlete, and to agree to participate in the study. Those athletes who did not meet the criteria above were excluded from the study. Athletes and their legal guardians (parent or trainer) who consented to participate in the study were provided with comprehensive information about the nature and scope of the investigation. They were asked to sign a written informed consent form approved by the ethics committee. A total of 201 licensed athletes participating in club teams across various sports branches completed the questionnaire.

### Questionnaire

The WHO Global Youth Tobacco Survey was adapted into a self-administered questionnaire for this study^16^. Before administering this questionnaire to the participants, a pre-test study was conducted and the questionnaire form was finalized. The respondents had to complete the questionnaire independently in an electronic format under the supervision of the researchers. The respondents were provided with comprehensive information regarding the specific content of the questionnaire and the definition of various types of tobacco products (cigarettes, rolled cigarettes, cigars, pipes, heated tobacco products and electronic cigarettes and waterpipe, etc.) and passive smoking.

The questionnaire asked about the use of tobacco and tobacco products. Those who reported smoking within the past 30 days were classified as current smokers who smoked cigarettes for one or more days in the past 30 days. Passive exposure to tobacco smoke was assessed both inside and outside the respondents’ homes. General exposure refers to the overall presence of tobacco smoke in environments frequented by the individual, such as public places, workplaces, and social settings. Daily exposure, on the other hand, focuses on routine and consistent exposure to tobacco smoke, particularly at home and in immediate surroundings. This comprehensive approach ensures a thorough understanding of the individual’s passive exposure to tobacco products and daily exposure to tobacco products inside and outside the home. Exposure inside the home was defined as at least one day of passive smoking by other people (parents, siblings, friends, and other individuals) at home in the last seven days. Exposure outside the home was defined as at least one day of passive smoking in places outside the home (cafes, coffee shops, tea houses, or training or competition venues) in the last seven days. The views of the respondents’ parents and siblings on the subjects of tobacco use, the acceptability of tobacco use at home, and the prohibition of tobacco use in public places were also elicited.

Data on the respondents’ age (years), and sports age (years) were collected by self-report. Weight, height, and waist circumference were measured by the researchers. Body weight was measured using a portable digital scale, and height was measured using a portable stadiometer. Waist circumference was measured as a point midway between the lowest edge of the rib cage and the highest edge of the iliac crest in a horizontal plane using non-elastic tape.

### Statistical analysis

Descriptive statistics were used to summarize the characteristics of the study participants. Continuous variables, such as age and sports age, are reported as mean and standard deviation (SD). Categorical variables, including tobacco use status, passive smoke exposure, and parental smoking status, are reported as frequencies and percentages. All analyses were performed using Jamovi software (version 2.3.0). The Shapiro-Wilk test was used to examine the fit of collected data to a normal distribution. Differences in the prevalence of active and passive tobacco smoking by gender were examined using the χ^2^ test. The significance level was set at 0.05. All tests were two-tailed.

## RESULTS

Of the athlete respondents, 36.3% (n=73) were female, and 63.7% (n=128) male. The average respondent was aged 13.4 ± 0.5 years. There were 36.3% (n=73) primary school students and 63.7% (n=128) secondary school students aged 13–14 years. The sports branches were football (n=86; 38.8% male, 4.0% female), taekwondo (n=31; 6.0% male, 9.5% female), volleyball (n=30; 1.5% male, 13.4% female), boxing (n=15; 5.5% male, 2% female) and basketball (n=9; 3.5% male, 1.0% female). On average, the respondents weighed 55.1 ± 11.6 kg, were 164.8 ± 9.0 cm tall, and had a waist circumference of 70.6 ± 10.4 cm. The mean sports age was 3.2 ± 2.0 years. More details on the demographic characteristics of the athletes are provided in Table 1.

**Table 1:** Demographic characteristics of the respondents, a cross-sectional study among adolescent athletes aged 13–14 years in Ankara province, Türkiye, 2023 (N=201)

*Characteristics*	*Mean ± SD*	*Min*	*Max*
**Age** (years)	13.4 ± 0.5	13.0	14.0
**Body weight** (kg)	55.1 ± 11.6	32.0	93.0
**Height** (cm)	164.8 ± 9.0	137.0	187.0
**Waist circumference** (cm)	70.6 ± 10.4	40.0	116.0
**Sports age** (years)	3.2 ± 2.0	1.0	8.0

**Figure 1:**
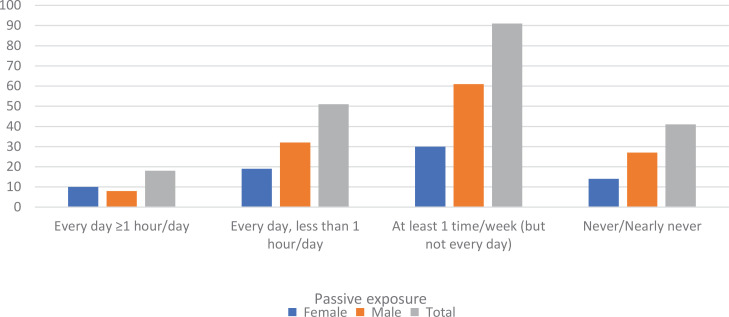
Passive exposure status of the respondents overall and by gender

In terms of tobacco use prevalence, 92.0% (n=185) of the athletes never used tobacco, 7.0% (n=14) tried it, 0.5% (n=1) quit using tobacco, and 0.5% (n=1) are currently using tobacco. A total of 20.4% (n=41) of the athletes indicated that they were passively exposed to tobacco smoke. Of the remaining athletes, 9% (n=18) reported that they had secondhand exposure to tobacco smoke for ≥1 h every day, 25.3% (n=51) for <1 h every day, and 45.3% (n=91) at least once a week. When asked about frequency, 34.3% (n=69) of the respondents reported daily and 45.3% (n=91) reported at least once a week secondhand exposure to tobacco smoke (Table 2, Figure 1).

**Table 2:** Active tobacco use and passive exposure status in general and by gender, a cross-sectional study among adolescent athletes aged 13–14 years in Ankara province, Türkiye, 2023 (N=201)

*Characteristics*	*Total* *n (%)^a^*	*Female* *n (%)^b^*	*Male* *n (%)^b^*	*χ²*	*p*
**Tobacco status**					
Never used	185 (92.0)	66 (35.7)	119 (64.3)	15.2	**<0.001**
Tried but not using	14 (7.0)	6 (42.9)	8 (57.1)	0.3	0.593
Quit	1 (0.5)	1 (100)	0 (0)		⁋
Daily	1 (0.5)	0 (0)	1 (100)		⁋
**Passive exposure**					
Every day ≥1 h/day	18 (9.0)	10 (55.6)	8 (44.4)	0.2	0.637
Every day <1 h/day	51 (25.3)	19 (37.3)	32 (62.7)	3.3	0.069
At least 1 time/week (but not every day)	91 (45.3)	30 (33.0)	61 (67.0)	10.6	**0.001**
Never/nearly never	41 (20.4)	14 (34.1)	27 (65.9)	4.12	**0.042**

a Column percentage.

b Row percentage.

⁋ Not calculated because the number of observations is 1.

The rates of secondhand smoking for one hour or more every day and less than one hour every day did not significantly differ by gender (p>0.05). However, passive exposure at least once a week was found to be significantly higher in male athletes (male: 67.0% vs female: 33.0 %; p<0.05), while no exposure was also found to be significantly higher in male athletes (female: 34.1% and male: 65.9%) (p<0.05) (Table 2). As reported in Table 3, 59.2% (n=119) of the respondents reported passive exposure to tobacco smoke inside and 71.1% (n=143) outside the home in the past 7 days.

**Table 3:** Passive exposure to tobacco smoke among the respondents inside and outside the home in the last 7 days, a cross-sectional study among adolescent athletes aged 13–14 years in Ankara province, Türkiye, 2023 (N=201)

*Exposure*	*Inside the home exposure n (%)*			*Outside the home exposure n (%)*		
	*Female*	*Male*	*Total*	*Female*	*Male*	*Total*
1–2 days/week	6 (3.0)	23 (11.4)	29 (14.4)	28 (13.9)	46 (22.8)	74 (36.7)
3–4 days/week	10 (5.0)	10 (5.0)	20 (10.0)	6 (3.0)	14 (7.0)	20 (10.0)
5–6 days/week	4 (2.0)	4 (2.0)	8 (4.0)	4 (2.0)	12 (6.0)	16 (8.0)
Every day	24 (11.9)	38 (18.9)	62 (30.8)	16 (8.0)	17 (8.4)	33 (16.4)
No one smoked	29 (14.4)	53 (26.4)	82 (40.8)	19 (9.5)	39 (19.4)	58 (28.9)
Total	73 (36.3)	128 (63.7)	201 (100)	73 (36.4)	128 (63.6)	201 (100)

Concerning parents who smoked inside the home, 10.4% (n=21) of the respondents reported that their mothers smoked, 36.3% (n=73) reported that their fathers smoked, and 19.9% (n=40) reported that both their parents smoked. None of the parents smoked tobacco among 33.3% (n=67) of the respondents (Table 4). In terms of passive tobacco smoking, 27.9% (n=56) reported that their mothers, 49.8% (n=100) reported that their fathers and 12.5% (n=25) reported that their siblings smoked at home (Table 4). When looking at the smoking rules of the respondents’ homes, 23.4% of the respondents lived in homes where smoking was permitted, while 28.4% had a strict no-smoking policy. Yet, 38.8% of the respondents reported smoking was not permitted but could be allowed in certain circumstances.

**Table 4:** Smoking of parents and siblings at home among the respondents, a cross-sectional study among adolescent athletes aged 13–14 years in Ankara province, Türkiye, 2023 (N=201)

*Smoking at home*		*Female* *n (%)*	*Male* *n (%)*	*Total* *n (%)*
**Mother**	Almost every day	16 (8.0)	22 (10.9)	38 (18.9)
	Sometimes	8 (4.0)	10 (5.0)	18 (9.0)
	Never	48 (23.9)	95 (47.2)	143 (71.1)
	I don’t have a mother	1 (0.5)	1 (0.5)	2 (1.0)
**Father**	Almost every day	17 (8.5)	43 (21.4)	60 (29.9)
	Sometimes	18 (9.0)	22 (10.9)	40 (19.9)
	Never	33 (16.4)	57 (28.3)	90 (44.7)
	I don’t have a father	5 (2.5)	6 (3.0)	11 (5.5)
**Siblings**	Almost every day	2 (1.0)	5 (2.5)	7 (3.5)
	Sometimes	5 (2.5)	13 (6.5)	18 (9.0)
	Never	58 (28.9)	99 (49.1)	157 (78.0)
	I don’t have a sibling	8 (4.0)	11 (5.5)	19 (9.5)

When asked whether tobacco products should be prohibited in public spaces, 95.1% of the respondents responded affirmatively. The majority of the respondents who endorsed the proposition to ban tobacco products reported that they considered this course of action to be beneficial for human health. Among 5% of the respondents who thought otherwise, 3% believed the implementation of the ban would result in a reduction in work efficiency.

## DISCUSSION

In our study assessing tobacco use among adolescent athletes aged 13–14 years in Ankara, Türkiye, we have found negligible rates of active tobacco use and high rates of passive tobacco smoking. The rate of secondhand smoking was significantly higher among males. Specifically, we have found that 34.3% of the respondents had passive exposure to tobacco smoke daily, while 45.0% had passive exposure at least once a week. The prevalence of secondhand smoke estimated in our study was lower than the 55.9% reported in a global study of youth aged 12–15 years^14^.

Prior studies documented that the use of tobacco by parents, particularly mothers, was found to be associated with subsequent uptake of tobacco by young adolescents^14^. In our study, 10.4% of the respondents had their mothers, 36.3% their fathers, and 19.9% both their parents smoking tobacco, which provides insight into the high prevalence of passive exposure within the home environment and raises concerns about the risk of smoking initiation among studied young athletes.

The results of the GYTS (Global Youth Tobacco Survey) conducted in 2016–2017 indicate that approximately 18% of adolescents aged 13–15 years were found to be current tobacco users^1^. In another global study, the average prevalence of tobacco use in young adolescents aged 12–15 years was found to be 13%. The latest GYTS data on adolescents aged 13–15 years indicates a prevalence of tobacco use of 11.3% in boys and 6.1% in girls based on the consumption of at least one cigarette in the last 30 days^17^. A 2019 study conducted in Peru revealed that the prevalence of smoking among adolescents was 4.4% among females and 7.0% among males^8^. Furthermore, the prevalence of tobacco use has been reported to be higher in adolescents aged 14–15 years compared with those aged 12–13 years in the majority of countries^14^. A study conducted in Türkiye revealed that 21.72% of male students aged 14–18 years who enrolled in high schools in Amasya province were cigarette smokers in 2017^12^. A study conducted in Ankara among students aged 11–19 years revealed a smoking rate of 28.1%^13^. In the present study, tobacco use was observed in only one athlete among adolescent athletes aged 13–14 years. Significantly lower prevalence of active smoking among our survey respondents could be attributed to their active engagement in sports and their young 13–14 age. Participation in sports and younger age may act as a deterrent to the uptake of tobacco products.

The lowest total scores on the Illegal Behavior Scale were observed in Art-Sports and Religious Schools^13^. The low prevalence of smoking in sports high schools, as observed in our study, indicates that engagement in sports may act as a preventive and/or reduction factor for smoking among adolescents.

In one study, the prevalence of passive smoking at home was found to be 17.4% in Mexico, 73.1% in Vietnam, 16.9% in Uruguay, and 65.8% in Bangladesh^18^. In another study, the prevalence of passive exposure at home was 14.0%, while the prevalence of passive exposure outside the home was 39.4%^8^. A study encompassing 15 countries, including Türkiye, demonstrated that passive exposure at home diminished as socioeconomic status increased^18^. Despite the implementation of smoke-free legislation, the prevalence of passive exposure both inside and outside the home remained considerable^8^. In the present study, the prevalence of passive exposure inside the home in the last 7 days was 59.2% and 71.1% outside the home. To reduce high passive exposure at home, periodic education and awareness-raising campaigns should be organized by experts in tobacco control. This can be achieved by inviting household members to primary healthcare facilities or by conducting home visits and educating them about the dangers of smoking. The education should provide information and raise awareness about quitting and combating addiction. Additionally, the number of free Tobacco Cessation Services should be increased.

A study conducted in China revealed that 74.8% of adolescents (aged 13–18 years) were exposed to tobacco smoke passively, with 36.6% of this group exposed both at home and in public places^19^. Given that 34.3% of the athletes in the present study were passively exposed to tobacco smoke daily, it is reasonable to conclude that a proportion of these young people will become active tobacco users in the future. As studies have demonstrated, passive exposure elevates the likelihood of future active tobacco use and induces tobacco sensitization^7,8,14^. Furthermore, it has been documented that the likelihood of e-cigarette utilization among students is 2.25 times greater with elevated levels of passive smoking than without it^19^.

The findings of our study align with prior research, including a study on Peruvian adolescents that reported low active smoking rates but significant passive smoke exposure, particularly at home^8^. Similarly, a study conducted in Indonesia found that passive smokers supported stronger tobacco control measures, as 95.1% of our adolescent athletes favored prohibiting public tobacco use^20^. Other studies have further highlighted the adverse effects of passive smoking, such as reduced lung function, impaired athletic performance, and developmental issues in adolescents^21-23^. These consistent findings underscore the widespread public health concern of passive smoking across demographics, emphasizing the need for targeted interventions, stricter smoke-free policies, and awareness campaigns, particularly in residential settings, to protect adolescents from secondhand smoke and its long-term risks.

The results of studies conducted on young people indicate that there is a discrepancy in the findings related to passive smoking. The results of the GYTS, conducted in 168 countries between 1999 and 2008, indicate that 30.4% of adolescents who had never smoked were exposed to passive tobacco smoke inside the home and 44.2% outside the home. Additionally, approximately 14% of adolescents had both parents and peers who were smoking. The most significant factor influencing passive smoking was the presence of parents or peers who were smoking^24^. A study conducted in Wuhan, China, in 2019, revealed that the prevalence of passive exposure at home, school, and public places among adolescents who had never smoked was 25.7%, 31.9%, and 48.9%, respectively. It has been demonstrated that the smoking habits of parents, peers, and teachers are significantly associated with the passive exposure of adolescents^25^. In 2016, a study conducted in Malaysia revealed that 37.8% of school-going adolescents aged 11–19 years had been exposed to secondhand smoke at home, 51.2% outside the home, and 27.3% at school over the last seven days^26^. A large-scale global study investigating passive exposure among adolescents conducted between 2010 and 2018, revealed a decline in the prevalence of passive exposure at home. However, the prevalence of passive exposure to tobacco smoke in public places has either increased or did not change in the majority of countries^27^.

Our study revealed that 23.4% of athletes’ homes permitted smoking, while the rate of athletes’ homes where smoking is not allowed save for some exceptional cases was found to be 38.8%. Among the athletes, 27.9% reported that their mothers, 49.8% reported that their fathers, and 12.5% reported that their siblings smoked at home.

The findings of our study indicate that adolescents engaged in sports in Türkiye are subject to a considerable degree of passive tobacco smoke exposure. It would be remiss to ignore the possibility that these adolescents may become active tobacco users in adulthood. The observation that the smoking prevalence among individuals aged ≥15 years in Türkiye in 2022 is as high as 28.3% indicates that passive exposure during adolescence is a significant risk factor for active tobacco use in adulthood^28^. In our study, 95.1% of the athletes responded affirmatively to the question of whether tobacco and tobacco products should be prohibited in public spaces. This indicates that adolescents are receptive to and supportive of the enactment of smoke-free legislation.

Both morbidity and mortality caused by active tobacco use and passive smoking are preventable. The implementation of smoke-free legislation has been demonstrated to markedly diminish the prevalence of smoking initiation among young people^29^. It is of great importance to protect adolescents from passive smoking, particularly in the home and in public places, in order to prevent the initiation of smoking in this age group.

There are many types of tobacco products. Nil prevalence of active smoking in this age group does not guarantee nil prevalence of smokeless tobacco use, for example, as adolescents get older. The respondents in this study also have a high probability of using tobacco in the future as a result of sensitization to smoking. Future studies can examine trajectories of tobacco use among adolescent athletes over their lifetimes.

### Strengths and limitations

The strengths of our study are the large sample size and the focus on a specific population group such as licensed adolescent athletes. However, the study has several limitations. First, the cross-sectional design prevents causal inferences; longitudinal studies are needed to explore temporal relationships. Second, the reliance on self-reported data for tobacco use may introduce bias. Nevertheless, we believe that the respondents provided accurate responses regarding their parents’ smoking habits and exposure to secondhand smoke outside their home. Objective measures, such as cotinine levels, could have improved reliability. Third, residual confounding from unmeasured factors like socioeconomic status or peer influences may have affected the results. Fourth, findings are limited to licensed athletes aged 13–14 years in specific districts, reducing generalizability to non-athletes or other regions. Including a non-athlete comparison group would have clarified the role of sports participation.

## CONCLUSIONS

Our findings indicate that active tobacco use is minimal among adolescent athletes aged 13–14 years in Ankara, Türkiye, potentially due to their engagement in sports and younger age group. However, passive exposure to tobacco smoke is prevalent, with over half of the athletes reporting exposure both inside and outside the home. Parental smoking was also common, with nearly half of the athletes having at least one parent who smokes. These results highlight the need for further attention to reducing passive smoke exposure among this population. Additional research is needed to explore the long-term health implications of passive smoke exposure and its potential influence on future tobacco use behaviors.

## Data Availability

The data supporting this research are available from the authors on reasonable request.
